# Response to: Comment on “Symptomatic Trifascicular Block in Steinert's Disease: Is It Too Soon for a Pacemaker?”

**DOI:** 10.1155/2016/4372802

**Published:** 2016-10-10

**Authors:** Glenmore Lasam, Roberto Roberti, Gina LaCapra, Roberto Ramirez

**Affiliations:** ^1^Department of Internal Medicine, Overlook Medical Center, Summit, NJ 07901, USA; ^2^Section of Cardiology, Overlook Medical Center, Summit, NJ 07901, USA

In acknowledgment to the “Comment on ‘Symptomatic Trifascicular Block in Steinert's Disease: Is It Too Soon for a Pacemaker?'” [1], below are the responses to the queries for clarification.

Steinert's disease or myotonic dystrophy type 1 (DM1) has been associated with the presence of an abnormal expansion of a CTG trinucleotide repeat on chromosome 19q13.3 [2] and has been correlated with cardiac involvement [3]. On our clinical vignette entitled “Symptomatic Trifascicular Block in Steinert's Disease: Is It Too Soon for a Pacemaker?” [4], we tried to retrieve the specific genetic code and expansion repeats for our patient but to no avail. The patient's neurologist retired a decade ago and we were unable to get the specific information about the genetics of our patient's disease. This concern had been raised by the editor prior to the approval of the manuscript. Family members were also contacted and were out of state and not living within the patient's locality anymore and were unwilling to divulge further information about their medical illnesses.

With regards to the patient's mental status, he has been alert, oriented, and coherent with no signs of any memory lapses or confusion. No further neurologic investigation was done since the dizziness improved significantly after the insertion of the pacemaker.

His pacemaker has been checked every six months and has no recorded ventricular arrhythmias. Also, there has been no elucidated significant history of cardiac disease in the family including sudden cardiac death.

The patient did not have frontal balding, cataracts, or myopathic face but has distal weakness and mild wasting as well as dysphagia compatible with esophageal dysmotility as documented on fluoroscopic barium esophagogram and upper gastrointestinal series.

As much as we want to include every detail of pertinent information in our clinical vignette, it has been restricted by the inability to extract the exact information from remote medical records and significant others' inability to share relevant information.

Truly, the relevance of such an interesting case could be increased by providing detailed clinical, genetic, and familial historical information to comprehensively investigate DM1 patients and their families to clear the enigma associated with this disease.
